# Pharmacogenomics of risperidone in autism spectrum disorder: a minireview

**DOI:** 10.3389/fphar.2026.1768109

**Published:** 2026-02-09

**Authors:** Caroline Rafaelli de Lima Honório, Beatriz Gafanhão Bobadilha, Melina Pinheiro Conscetta, Felipe De Mello Silveira, Francisco Durigon, Aline Cristiane Planello

**Affiliations:** Department of Morphology and Basic Pathology - Medical School, Faculdade de Medicina de Jundiaí, Jundiaí, Brazil

**Keywords:** autism spectrum disorder, *CYP2D6*, personalized medicine, pharmacogenomics, risperidone

## Abstract

Risperidone is one of the most widely prescribed antipsychotics for the management of irritability and associated behavioral symptoms in autism spectrum disorder (ASD), yet clinical response and adverse-effect risk vary widely among individuals. Pharmacogenomic (PGx) research has sought to explain this variability, with accumulating evidence pointing to contributions from metabolic, transporter, and neurotransmitter pathways. In this narrative minireview, we synthesize current findings on PGx factors influencing risperidone outcomes in children and adolescents with ASD. *CYP2D6* emerges as the most robust predictor of pharmacokinetics and toxicity, while pharmacodynamic associations involving dopaminergic, serotonergic, and metabolic pathways in genes such as *ABCB1*, *DRD3*, *HTR2A*, *HTR2C*, and *LEP* remain inconsistent and largely derived from small cohorts. We also discuss methodological challenges in assessing treatment response, current clinical guidelines, barriers to implementation, and emerging approaches including polygenic models, pharmacoepigenomics, and machine learning. Together, the available evidence points to both the promise and the limitations of PGx in guiding safer and more individualized risperidone therapy in ASD.

## Introduction

Autism spectrum disorder (ASD) is diagnosed in approximately 0.6%–1% of children around the world and is characterized by persistent deficits in social communication alongside restricted, repetitive patterns of behavior (Zeidan et al., 2022). Among the most challenging aspects of ASD management are the behavioral comorbidities, including irritability, aggression, and self-injurious behaviors, which significantly impact quality of life for both individuals with ASD and their families (Maneeton et al., 2018). Risperidone, an atypical antipsychotic, represents one of only two FDA-approved medications for treating irritability associated with ASD in children and adolescents aged 5–17 years (Nagaraj et al., 2006).

The clinical landscape of risperidone therapy in ASD is marked by considerable heterogeneity in treatment outcomes. While randomized controlled trials consistently demonstrate efficacy compared to placebo, with effect sizes ranging from 0.7 to 1.2 on standardized behavioral measures (Rossow et al., 2021), real-world clinical experience reveals substantial interindividual variability. Approximately 60%–70% of patients experience clinically meaningful improvement, while the remaining 30%–40% show minimal response or develop intolerable adverse effects including significant weight gain, metabolic dysfunction, and hyperprolactinemia (Sukasem et al., 2018). This variability has catalyzed extensive research into pharmacogenomic (PGx) factors that might predict treatment outcomes and guide personalized therapy approaches (Biswas et al., 2022).

The field of PGx, which examines how genetic variations influence drug response, has evolved considerably over the past decade in pediatric populations (Alshabeeb et al., 2022). For risperidone, research has progressed from small candidate gene association studies toward moderately sized cohorts and broader analytic frameworks incorporating panels of drug-metabolizing enzymes and transporters, such as DMET-based approaches (Medhasi et al., 2016). However, despite accumulating evidence, translation of PGx findings into routine clinical practice remains limited, particularly in pediatric neurodevelopmental populations such as children and adolescents with ASD (Biswas et al., 2023).

This narrative review synthesizes current knowledge on the pharmacogenomics of risperidone in ASD. We examine the mechanistic basis of pharmacogenomic effects, review evidence for specific genetic variants, discuss challenges related to clinical outcome assessment, and evaluate current guidelines and barriers to implementation. In addition, we highlight emerging approaches, including pharmacoepigenomics, polygenic modeling, and machine-learning–based integrative frameworks, that may help address the limitations of current candidate-gene studies and advance more precise treatment strategies for ASD.

## Pharmacology of risperidone in ASD

Risperidone’s therapeutic effects in ASD are primarily attributed to dopamine D2 receptor antagonism, which modulates mesolimbic dopaminergic signaling implicated in behavioral dysregulation, impulsivity, and aggression (Janssen et al., 1988; Research Units on Pediatric Psychopharmacology Autism Network, 2005; Nagaraj et al., 2006). In children and adolescents with ASD, excessive irritability and aggressive outbursts are thought to reflect, at least in part, altered dopaminergic tone within fronto-striatal and limbic circuits involved in behavioral control and emotional regulation (McCracken et al., 2002; Canitano and Scandurra, 2008). By attenuating dopaminergic signaling in these pathways, risperidone reduces the frequency and severity of disruptive behaviors, including aggression, self-injury, and severe irritability, which constitute its main approved indications in ASD.

In addition to dopamine D2 blockade, risperidone antagonizes serotonin 5-HT2A receptors, a property that modulates dopaminergic neurotransmission within cortico-striatal circuits and contributes to behavioral stabilization (McCracken et al., 2002). This dopaminergic–serotonergic interaction is considered a defining feature of second-generation antipsychotics and helps explain both the clinical efficacy of risperidone in managing irritability and its comparatively favorable extrapyramidal side-effect profile relative to first-generation agents. At the same time, dopamine D2 antagonism in tuberoinfundibular pathways is associated with prolactin elevation (Nagaraj et al., 2006), a clinically relevant adverse effect in pediatric populations, underscoring the narrow therapeutic window of risperidone in ASD. The drug also has affinity for α1-and α2-adrenergic receptors and H1-histaminergic receptors (Corena-McLeod, 2015), contributing to sedation, orthostatic hypotension, and weight gain. The combined activity across dopaminergic, serotonergic, and histaminergic pathways is thought to shape both therapeutic response and side-effect profiles, underscoring the relevance of interindividual variability in receptor sensitivity and downstream signaling.

Pharmacokinetically, risperidone is metabolized mainly by CYP2D6, which converts it to 9-hydroxyrisperidone (paliperidone), an active metabolite with similar receptor affinity (Janssen Pharmaceutical Companies, 2025). Plasma concentrations of the combined “active moiety” vary considerably across individuals due to CYP2D6 genotype, age, co-medications, and metabolic capacity. This metabolic pathway represents the most significant source of pharmacokinetic variability and the primary target of PGx investigation.

## Pharmacogenomics of risperidone in ASD

Interindividual variation in risperidone response among ASD population has long suggested a genetic contribution, even though the underlying architecture remains only partially resolved (Medhasi et al., 2016; Brown et al., 2017; Sukasem et al., 2018; Hongkaew et al., 2021; Alshabeeb et al., 2022; Biswas et al., 2023). Most available data come from modestly sized candidate-gene studies, yet several reproducible signals have emerged, particularly in genes involved in metabolism and serotonergic or dopaminergic signaling. Although the evidence base is uneven, patterns across cohorts allow cautious interpretation and point toward a polygenic and mechanistically diverse landscape (Shilbayeh et al., 2024).

To contextualize the current evidence base, Table 1 summarizes the most frequently investigated PGx variants related to risperidone response in ASD, including metabolic, transporter, neurotransmitter, and neuroendocrine pathways. The table compiles findings across diverse cohorts and highlights the heterogeneity of evidence strength, with *CYP2D6* representing the only gene reaching high-confidence classification (PharmGKB level 1A -https://www.clinpgx.org/). In fact, the most consistent predictor of risperidone pharmacokinetics and adverse effects is *CYP2D6*. Across multiple populations, poor metabolizers (PMs) exhibit higher plasma concentrations and a 2–3-fold increased risk of dose-dependent adverse events, notably prolactin elevation and weight gain (Rossow et al., 2021). Interestingly, efficacy differences are subtle as PMs may still improve clinically, albeit sometimes at lower dose, reinforcing that the primary impact lies in toxicity risk rather than therapeutic failure. Metabolic phenotype influences toxicity far more reliably than efficacy, reinforcing *CYP2D6* as the only pharmacogene with replicated effects across ASD cohorts.

**TABLE 1:** Summary of pharmacogenomic variants studied in relation to risperidone response and adverse effects in Autism Spectrum Disorder (ASD).

Gene	Variant(s)	Reported association	Study populations (N)	Evidence summary	PharmGKB evidence level for ASD	References
*CYP2D6*	PM alleles (e.g., *4, *5, *10, etc.): cause reduced enzyme activity	↑ adverse effects (2–3× risk), ↑ prolactin, ↑ weight gain; minimal impact on efficacy	USA (257); Thailand (84); Portugal (45); Saudi Arabia (83); Switzerland (515); Nigeria (208); Israel (40)	Most replicated marker; strong predictor of PK and toxicity	1A	Correia et al. (2010), Youngster et al. (2014), Vanwong et al. (2016), Rossow et al. (2021), Shilbayeh et al. (2024), Kehinde et al. (2025), Piras et al. (2025)
*ABCB1*	Synonymous (1236C>T,rs1128503).Also studied: 2677G>T/A, 3435C>T	T allele: possible ↑ response; no clear toxicity effect	Portugal (45); Thailand (134)	Mixed findings; small effect size; needs replication	3	Correia et al. (2010), Vanwong et al. (2020)
*BDNF*	Val66Met (c.196G>A, rs6265)	Met allele: ↑ prolactin; possible insulin resistance; no efficacy effect	Portugal (45); Thailand (89)	Two small studies; needs replication	-	Correia et al. (2010), Sukasem et al. (2018)
*CNR1*	Promoter (c.-63-4495G>A, rs806378); Synonymous (c.1359G>A, rs1049353)	↑ weight gain risk	USA (181)	Single ASD cohort; biologically plausible	3	Nurmi et al. (2013)
*DRD2 (ANKK1)*	Taq1A (c.2137G>A, rs1800497)	A allele: nonstable response; diplotypes: ↑ prolactin	Thailand (124, 82); Spain (100);	Mixed finding: more data needed	-	López-Rodríguez et al. (2011), Nuntamool et al. (2017)
*DRD3*	Ser9Gly (c.25G>A, rs6280)	Gly carriers:↑ treatment efficacy	Portugal (45); Iran (56)	Replicated in two studies: promising	3	Correia et al. (2010), Firouzabadi et al. (2017)
*HTR2A*	promoter (−329 + 609G>A, rs6311; in LD with T102C)	A allele: ↑ improvement; ↓ AE	Portugal (45); USA (257); Saudi (89)	Consistent directionality	3	Correia et al. (2010), Rossow et al. (2021), Shilbayeh et al. (2024)
*HTR2C*	Cys23Ser (c.68G>C, rs6318); −759C>T (rs3813929)	Ser23: ↑ metabolic risk; T allele: ↑ efficacy	Portugal (45); Thailand (134); Saudi (89)	Mixed findings, but biologically coherent	3	Correia et al. (2010), Rossow et al. (2021), Shilbayeh et al. (2024)
*HTR6*	Intronic SNP (c.7154–2542C>T)	↑ prolactin	Portugal (45)	Single study; limited evidence	-	Correia et al. (2010)
*LEP*	promoter (c.-2605G>A, rs7799039)	G allele: ↑ weight gain	Thailand (89); USA (181)	Replicated in two studies; low effect size; promising	3	Nurmi et al. (2013), Sukasem et al. (2018)
*TYMS*	Synonymous (c.381A>G, rs3786362)	↑ prolactin	Thailand (84)	Exploratory; not established	-	Hongkaew et al. (2018)
*UGT1A1*	UGT1A128 haplotype (promoter repeats) c.-364C>T, c.-3156G>A, c.-2950A>G variants in complete LD.	↑ prolactin (reduced function)	Thailand (84)	Exploratory; biologically plausible	3	Hongkaew et al. (2018)

Abbreviations: ASD, autism spectrum disorder; PM, poor metabolizer; PK, pharmacokinetics; AE, adverse events; LD, linkage disequilibrium.

Although CYP2D6 activity is low at birth, clinical and phenotyping studies indicate that enzymatic activity approaches adult levels within the first year of life and remains stable throughout childhood and adolescence, with minimal modulation by age or pubertal development (Blake et al., 2007; Nofziger et al., 2020; Leeder et al., 2022). In line with this developmental profile, population pharmacokinetic analyses of risperidone show that, after adjustment for body weight, exposure to risperidone and its active metabolite is comparable between children, adolescents, and adults, while CYP2D6 metabolizer status remains the primary determinant of plasma concentrations and adverse-effect risk (Thyssen et al., 2010).

Transporter and receptor genes show more heterogeneous associations. *ABCB1* variants have been linked to differential response in a cohort (Correia et al., 2010), likely through modulation of blood–brain barrier efflux, although findings remain inconsistent (Vanwong et al., 2020). Likewise, *CNR1* promoter variants and *LEP* polymorphisms have been associated with weight-gain susceptibility effects (Nurmi et al., 2013) that appear biologically plausible but require replication.

Genes involved in dopaminergic signaling, including *DRD2/ANKK1* and *DRD3*, have produced some of the clearest pharmacodynamic signals. *DRD3* Ser9Gly (rs6280) has been linked to enhanced clinical improvement in two independent studies (Correia et al., 2010; Firouzabadi et al., 2017). By contrast, *DRD2/ANKK1* Taq1A has shown associations with non-stable response patterns (Nuntamool et al., 2017) or prolactin elevations (López-Rodríguez et al., 2011) rather than consistent efficacy outcomes.

Serotonergic genes, particularly *HTR2A* and *HTR2C*, also demonstrate preliminary but intriguing associations. *HTR2A* rs6311 has been tied to greater behavioral improvement and fewer adverse effects (Correia et al., 2010; Rossow et al., 2021; Shilbayeh et al., 2024), whereas *HTR2C* −759T and Cys23Ser variants influence both symptom reduction and metabolic liability across some cohorts. *HTR6* and *UGT1A1* variants have been associated with risperidone-induced hyperprolactinemia in small sample size (Hongkaew et al., 2018), although validation is still lacking.

Overall, pharmacodynamic associations reported for risperidone response in ASD remain based on relatively small cohorts, in part because clinical efficacy and longitudinally monitor is inherently more difficult to quantify than pharmacokinetic endpoints such as plasma drug levels. Much of the available evidence is derived from European studies, most notably from Portugal, and from Asian populations, particularly cohorts from Thailand. While this geographic distribution may be viewed as an early and encouraging step toward investigating PGx effects beyond traditionally overrepresented Western European and North American populations, the limited sample sizes and lack of independent replication constrain the generalizability of these findings. Robust validation in larger, ancestrally diverse cohorts is still needed to determine whether reported associations reflect true biological effects or context-specific signals. Addressing this gap will be essential for translating pharmacodynamic insights into clinically meaningful guidance for heterogeneous ASD populations.

## Clinical assessment and pharmacogenomic correlations

Evaluating risperidone response in ASD presents challenges that extend beyond standard psychopharmacology. The marked clinical heterogeneity of the disorder, well documented across diagnostic instruments such as ADOS and ADI-R (Lebersfeld et al., 2021), creates an additional layer of complexity when defining what constitutes treatment response. This variability supports the need for outcome measures capable of capturing both behavioral change and functional adaptation. Although gold-standard diagnostic tools provide highly structured assessments of core ASD features, they are rarely incorporated into pharmacologic trials because they are not optimized for detecting short-term behavioral change (Matson et al., 2007).

The Aberrant Behavior Checklist–Community version (ABC-C) remains the most widely used instrument in risperidone trials, with the Irritability subscale serving as the primary endpoint (Aman et al., 1985; Arnold et al., 2003; Shea et al., 2004). Its sensitivity to reductions in aggression, tantrums, and self-injury has made it the field’s *de facto* standard. Still, the ABC-C centers on associated behaviors rather than core ASD features, which may obscure more nuanced domains of improvement, an issue that becomes increasingly relevant when examining genotype-phenotype correlations (Smith, 2017). For instance, pharmacokinetic variants influencing tolerability may indirectly affect ABC-I scores by constraining dose escalation rather than altering behavioral mechanisms *per se* (Biswas et al., 2023).

The Clinical Global Impression (CGI) provides a broader, clinician-anchored evaluation of improvement and severity (Busner and Targum, 2007). Because CGI scores integrate both symptomatic change and functional impression, they sometimes appear more sensitive to adverse events tied to PGx variation, for example, in patients with CYP2D6 poor-metabolizer phenotypes who show dose-limiting side effects (Collins et al., 2020). Yet, the CGI’s subjectivity also introduces variability that can mask subtle genomic associations (Lu et al., 2021).

In contrast to these short-term behavioral measures, adaptive-functioning instruments such as the Vineland Adaptive Behavior Scales (VABS) offer a broader view of communication, socialization, and daily-living skills (Villa et al., 2010). Although VABS has been incorporated into some pharmacologic studies, including risperidone extensions (Kim et al., 2022), it is rarely used as a primary endpoint, limiting its current relevance for PGx analyses.

The Verbal Behavior Milestones Assessment and Placement Program (VB-MAPP) provides another complementary perspective by offering structured metrics of verbal, pre-academic, and adaptive milestones (Dixon et al., 2015). Despite its widespread use in behavioral interventions, VB-MAPP has not yet demonstrated sensitivity to short-term medication effects or been used in PGx. Even so, its structured developmental metrics could, in principle, serve as valuable phenotypes in future PGx studies, particularly for capturing downstream functional gains not reflected in conventional behavioral scales.

Across instruments, a recurring theme is that phenotyping choices shape the detectability of PGx effects (Maranville and Cox, 2016; Smit et al., 2018). Variants influencing exposure and tolerability may manifest through caregiver reports of sedation, irritability reduction, or early discontinuation, whereas pharmacodynamic markers may express themselves more subtly in stabilization patterns over time. As PGxs moves toward multi-gene models, the precision of clinical assessment will become increasingly important, not simply to measure improvement but to define the dimensions of response most biologically relevant to genetic variation.

## Current guidelines and clinical implementation

Despite increasing interest in PGx-guided prescribing for antipsychotics, formal guidance specific to risperidone remains limited. The Clinical Pharmacogenetics Implementation Consortium (CPIC) is currently developing a risperidone–CYP2D6 guideline, reflecting growing recognition of the clinical relevance of CYP2D6-mediated variability (https://cpicpgx.org/prioritization-of-cpic-guidelines/). Although the final recommendations have not yet been released, they are expected to align with existing CYP2D6-based frameworks, with implications for dose optimization in poor metabolizers and enhanced monitoring for adverse effects. Regulatory agencies similarly acknowledge the clinical relevance of *CYP2D6* for risperidone pharmacokinetics: FDA clinical pharmacology data demonstrate substantially higher active-moiety exposure in *CYP2D6* PMs, consistent with reduced metabolic clearance, yet the approved label does not mandate genotyping nor provide genotype-based dosing recommendations (Janssen Pharmaceutical Companies, 2025). To date, the Dutch Pharmacogenetics Working Group offers the most explicit guidance, recommending lower starting doses or slower titration in *CYP2D6* PMs (Beunk et al., 2024). However, these recommendations are derived primarily from adult psychiatric populations, and their applicability to children with ASD, who differ in developmental pharmacokinetics, comorbidity profiles, and vulnerability to adverse effects, remains uncertain. Key differences among DPWG, FDA labeling, and CPIC with respect to CYP2D6-guided dose adjustment for risperidone are summarized in Table 2.

**TABLE 2:** CYP2D6-guided dosing recommendations for risperidone across DPWG, FDA, and CPIC frameworks.

Guidelines	Adjustment for PM (poor metabolizers)	Adjustment for UM (ultrarapid metabolizers)
DPWG	Yes (33%–50% dose reduction)	Yes (alternative drug or titration based on active metabolite)
FDA	No (standard clinical adjustment)	Not explicitly mentioned in label
CPIC	In development (Provisional Level B)	In development (Provisional Level B)

The table compares guidance from the Dutch Pharmacogenetics Working Group (DPWG), the U.S., Food and Drug Administration (FDA) labeling, and the Clinical Pharmacogenetics Implementation Consortium (CPIC) regarding dose adjustment in poor metabolizers (PM) and ultrarapid metabolizers (UM).

Professional societies have taken a cautious stance. The American Academy of Child and Adolescent Psychiatry explicitly advises against the routine clinical use of PGx testing for psychotropic prescribing in youth, citing insufficient evidence for clinical utility and substantial variability across commercial panels (AACAP Policy Statement, 2020). The American Psychiatric Association echoes this position, noting that current data do not support widespread implementation of PGx testing in psychiatric practice (Baum et al., 2024). This restraint reflects broader concerns within the field, including limited replication of pharmacodynamic associations, small sample sizes in ASD-focused cohorts, and the fact that *CYP2D6* remains the only risperidone-related pharmacogene with consistently validated clinical relevance.

## Implementation challenges and barriers

Moving PGx findings from research settings into routine clinical care remains a substantial challenge across multiple clinical domains, but this transition is particularly complex in pediatric populations with neurodevelopmental conditions. One major obstacle is the complexity of *CYP2D6* genotyping. The gene’s architecture features high homology with pseudogenes, frequent hybrid alleles, and copy number variation and requires specialized laboratory methods and careful interpretation (Nofziger et al., 2020; Taylor et al., 2020). Misclassification of metabolizer status is not merely theoretical; inaccurate copy-number calls can lead to clinically significant phenotype errors, which complicates the development of reliable decision-support tools (Nguyen et al., 2009; Nofziger et al., 2020). While long-read sequencing may eventually streamline haplotype resolution (Turner et al., 2023), these technologies remain cost-prohibitive for most clinical programs.

Integration into clinical workflows presents another layer of difficulty. Many electronic health record systems do not yet support seamless incorporation of genotype data or context-sensitive dosing alerts (Gross and Daniel, 2018; Almeida et al., 2021). Decision-support models that work well for adult populations must be recalibrated for pediatrics, where weight-based dosing, rapid developmental changes, and differential side-effect profiles introduce additional complexity (Virelli et al., 2021). In practice, clinicians often report uncertainty about how to interpret PGx results in the context of a child with ASD whose behavioral symptoms fluctuate and whose treatment goals may extend beyond simple symptom reduction (Yoshida et al., 2021).

Economic considerations further complicate adoption. PGx testing costs vary widely, and reimbursement policies remain inconsistent across insurance providers (Virelli et al., 2021). Although it is suggested that genotype-guided psychiatry prescribing could reduce adverse-event-related costs over time (Oslin et al., 2022), the initial investment is nontrivial, and cost-effectiveness models rarely incorporate the unique features of ASD care.

The ethical and social dimensions of genetic testing in children also warrant careful attention (Botkin et al., 2015). Parents must weigh potential benefits against concerns about genetic privacy, future insurability, and whether test results might raise questions unrelated to the immediate clinical scenario. These concerns can be more acute in ASD care, where families already navigate complex diagnostic pathways and multiple therapeutic modalities. Transparent communication and culturally sensitive counseling therefore become essential components of any implementation strategy (Pereira et al., 2024).

Finally, issues of population diversity represent a persistent challenge (Bianco and Planello, 2025). Most PGx data for risperidone derive from European or Asian ancestry groups, with comparatively fewer or no studies in African, Middle Eastern, or Latin American populations (Maggo et al., 2025). Given substantial interethnic differences in *CYP2D6* allele frequencies and metabolic profiles, genotype–phenotype relationships established in one population cannot be assumed to generalize to another (Ribeiro et al., 2024; Maggo et al., 2025). Emerging research cohorts has begun to address these gaps (Maggo et al., 2025), but the evidence base remains uneven, and the risk of exacerbating existing health disparities is non-negligible if implementation proceeds without representative data.

## Future directions and emerging approaches

Advances in neurogenomics and data science are beginning to reshape how PGx questions are framed in psychiatry, and risperidone treatment in ASD may eventually benefit from several of these emerging approaches. One promising direction involves polygenic risk scores (PRS). Although many currently available polygenic risk scores were derived from variants associated with disease susceptibility rather than pharmacological response (Singh et al., 2024), here is growing interest in evaluating whether polygenic liability to irritability, aggression, or metabolic dysregulation may modify clinical response or adverse-effect risk during risperidone treatment (De Pieri et al., 2024). Early conceptual models of antipsychotics in schizophrenia suggest that PRS might refine risk stratification beyond what *CYP2D6* alone can achieve, particularly in cases where pharmacodynamic pathways exert small but cumulative effects (Chen et al., 2018; Zhang et al., 2019). Still, the utility of PRS in pharmacotherapy remains speculative, and its implementation will require large, ancestrally diverse cohorts to avoid miscalibration (Singh et al., 2024).

A parallel development involves pharmacoepigenomics, which may help explain why individuals with similar genotypes can show markedly different clinical outcomes (Swathy and Banerjee, 2022). DNA methylation signatures associated with stress response, metabolic regulation, or serotonergic signaling have been proposed as modulators of antipsychotic sensitivity (Adanty et al., 2022; Du et al., 2022; Alvarado et al., 2025). In ASD specifically, where gene–environment interactions are particularly salient, epigenetic markers could offer an additional layer of biological context (Yan, 2025). Whether these signatures precede treatment, emerge as drug-induced adaptations, or reflect broader developmental conditions remains an open question, but their incorporation into PGx frameworks is a natural next step.

Meanwhile, machine-learning and integrative modeling approaches are redefining what is methodologically feasible in antipsychotic research, particularly within schizophrenia, where several recent studies have demonstrated substantial gains in predictive accuracy (Guo et al., 2025; Yee et al., 2025). Algorithms that combine clinical trajectories, genotypes, metabolizer phenotypes, and real-world medication adherence may detect patterns invisible to traditional statistical approaches (Comai et al., 2025). Translating these approaches to ASD holds clear promise as such models could eventually help differentiate true pharmacodynamic nonresponse from the behavioral variability inherent to neurodevelopmental conditions. The challenge, however, lies in ensuring interpretability, clinicians must understand why a model predicts nonresponse or elevated risk (Weitzel et al., 2014), particularly when treatment decisions involve vulnerable pediatric populations.

## Conclusion

PGx research on risperidone in ASD has advanced meaningfully in recent years, with *CYP2D6* emerging as the clearest and most actionable predictor of drug exposure and adverse-effect risk. Evidence for pharmacodynamic associations is growing but remains inconsistent, reflecting the small size and heterogeneity of available cohorts. Insights generated from larger antipsychotic studies in schizophrenia, particularly regarding pharmacodynamic pathways, epigenetic modulation, and integrative machine-learning frameworks, may offer a conceptual foundation for analogous investigations in ASD, provided that developmental and neurobiological differences are carefully considered. Even so, the convergence of genomic, clinical, and computational approaches points toward a future in which treatment decisions may be increasingly individualized. Real progress will depend on larger, ancestrally diverse pediatric studies and on implementation strategies that integrate genetic data into real-world care. Together, these efforts may transform risperidone prescribing from a trial-and-error process into a more precise and informed component of ASD management.
